# Generation of VEGF knock-in Cashmere goat via the CRISPR/Cas9 system

**DOI:** 10.7150/ijbs.55559

**Published:** 2021-03-02

**Authors:** Xiao Hu, Fei Hao, Xiaocong Li, Zhiyuan Xun, Yuan Gao, Bingxu Ren, Ming Cang, Hao Liang, Dongjun Liu

**Affiliations:** State Key Laboratory of Reproductive Regulation & Breeding of Grassland Livestock, Inner Mongolia University, Hohhot, 010000, China.

**Keywords:** CRISPR/Cas9, VEGF, knock-in, cashmere goats, hair growth

## Abstract

Cashmere is a rare and specialised animal fibre, which grows on the outer skin of goats. Owing its low yield and soft, light, and warm properties, it has a high economic value. Here, we attempted to improve existing cashmere goat breeds by simultaneously increasing their fibre length and cashmere yield. We attempted this by knocking in the vascular endothelial growth factor (*VEGF*) at the fibroblast growth factor 5(*FGF5*) site using a gene editing technology and then studying its hair growth-promoting mechanisms. We show that a combination of RS-1 and NU7441 significantly improve the efficiency of CRISPR/Cas9-mediated, homologous-directed repair without affecting the embryo cleavage rate or the percentages of embryos at different stages. In addition, we obtained a cashmere goat, which integrated the *VEGF* gene at the *FGF5* site, and the cashmere yield and fibre length of this gene-edited goat were improved. Through next-generation sequencing, we found that the up-regulation of *VEGF* and the down-regulation of *FGF5* affected the cell cycle, proliferation, and vascular tone through the PI3K-AKT signalling pathway and at extracellular matrix-receptor interactions. Owing to this, the gene-edited cashmere goat showed impressive cashmere performance. Overall, in this study, we generated a gene-edited cashmere goat by integrating *VEGF* at the *FGF5* site and provided an animal model for follow-up research on hair growth mechanisms.

## Introduction

The emergence and development of gene editing have realised the site-directed transformation of genomic DNA sequences and promoted a revolution in the field of biomedicine. A pre-condition for gene editing is the double-strand break (DSB). However, the efficiency of traditional gene manipulation technology to achieve gene editing using donor plasmids and naturally generated DSB is very low, i.e. approximately 10^-4^-10^-6^, which limits the application of this technology in gene function research, disease model construction, and genetic breeding 1-4. Therefore, we need to artificially introduce a DSB.

Over the past few decades, from the restriction enzymes of the 1970s to today's advanced gene-editing techniques, the precise manipulation of eukaryotic genomes with molecular tools derived from the prokaryotic immune system has improved. Most notable is the introduction of clustered regulatory interspaced short palindromic repeat (CRISPR) editing techniques 5-7. The CRISPR/CRISPR-associated protein-9 nuclease (Cas9) system is a third-generation gene-editing technology known for its unprecedented specificity, effectiveness, and versatility 8. It is derived from the acquired immune system of prokaryotes, providing acquired immunity against the invasion of foreign gene elements such as phages, and is widely found in archaea and other prokaryotes 9.

Compared with the traditional zinc-finger and transcription activator-like effector nucleases, the CRISPR/Cas9 system has higher gene-editing efficiency and lower production costs. It can be flexibly applied to different nucleotide sequences and is a more powerful gene-editing tool 10. With the emergence, upgradation, and improvement of CRISPR/Cas9, the efficiency of DSB generation in the genome has become fully guaranteed, which provides favourable conditions for the development of gene-editing animal models.

The Inner Mongolian Albas cashmere goat is an excellent local cashmere and meat hybrid breed formed through long-term natural breeding. The cashmere produced is of high quality and has a high economic value, but the cashmere yield is low. Therefore, it is very important to cultivate new cashmere goat lines with higher yields. However, the conventional breeding technology has several disadvantages, such as the need for a large initial investment, slow effects, and long cycle. The breeding of new varieties with high yields, high qualities, and other excellent traits is thus progressing relatively slowly. Therefore, gene-editing technology should be used to cultivate new cashmere goat strains with high yields.

Vascular endothelial growth factor (*VEGF*) is an active and widely distributed growth factor secreted by vascular endothelial cells. It can increase vascular permeability and plays an irreplaceable role in angiogenesis and mesenchymal cell differentiation 11-13. Studies have shown that, after binding to its receptor *VEGFR-2*, *VEGF* can promote hair growth by stimulating cell proliferation and regulating the hair growth cycle 14, 15.

Hair growth in mammals shows seasonal and periodic changes in anagen, catagen, and telogen content 16. Therefore, prolonging the anagen phase can improve the quality and yield of hair. The hair growth cycle is regulated by a series of cell growth factors 17. As fibroblast growth factor (FGF)-5 is a signalling protein secreted during the hair growth cycle, mutations in mammalian *FGF5* can prolong the anagen phase and lead to new long-hair traits 18-23.

It has been reported that embryos microinjected with CRISPR/Cas9 can be used to obtain mosaic goats with *FGF5* gene disruption, which increase the length of the cashmere fibres in these cashmere goats 24. However, the stable inheritance of qualified characteristics is essential in the genetic breeding of domestic animals. Therefore, in this study, we used somatic cell nuclear transfer (SCNT) to prepare a gene-edited cashmere goat which can transmit superior traits to the next generation.

In conclusion, we here attempted to combine CRISPR/Cas9 and SCNT to generate gene-edited cashmere goats that integrate *VEGF* at the *FGF5* site and promote *VEGF* overexpression through *FGF5* knockout. The results of this study provide a basis for the efficient generation of gene knock-in animals as well as an animal model for studying the hair growth-promoting mechanisms of cashmere goats.

## Materials and Methods

### Ethics statement

All experiments performed followed the National Research Council Guide for the Care and Use of Laboratory Animals. All protocols were approved by the Institutional Animal Care and Use Committee of Inner Mongolia University. All animals were maintained at the Inner Mongolia YiWei White Cashmere Goat Limited Liability Company.

### Cell culture

Goat foetal fibroblast cells (GFFs) were isolated from goat embryos at 40 days. The cells were maintained in Dulbecco's Modified Eagle's Medium (DMEM): nutrient mixture F-12 (Gibco, Carlsbad, USA), supplemented with 15% foetal bovine serum (FBS, Biological Industries, Kibbutz Beit Haemek, Israel). The culture was kept in an incubator with 5% CO_2_ at 37 °C. Cells in the log phase were seeded into six-well plates. The RAD51-stimulatory compound, RS-1 25 (10 μM, Selleck, Houston, USA), and the DNA-PKcs inhibitor, NU7441 26 (2 μM, Selleck), dissolved in dimethyl sulfoxide, were diluted with the cell culture medium.

### Cell cycle analysis

The cell cycle was measured using the Cell Cycle and Apoptosis Analysis Kit (7sea Biotech, Shanghai, China). Cell suspensions were prepared from GFFs treated with inhibitors in pre-cooled phosphate-buffered saline (PBS, Gibco). After an overnight fixation with 70% ethanol at 4 °C, the cells were stained with 500 μL of 2.5% propidium iodide (PI) solution at 37 °C for 30 min away from light. Flow cytometry (BD Biosciences, New York, USA) was performed within 5 h, and the excitation wavelength was 488 nm.

### Apoptosis analysis

Apoptotic cells were detected using the Annexin V-FITC/PI Apoptosis Analysis Kit (7sea Biotech). GFFs were inoculated in 24-well plates, in triplicate, and cultured for 24 h. After the addition of inhibitors, the culture was continued for 24 h. The culture solutions were then collected in centrifuge tubes because they contained apoptotic cells. Then, cells in each group were trypsinised and washed with PBS to completely remove the trypsin. Subsequently, the cells were re-suspended in 400 μL of binding buffer. V-fluorescein isothiocyanate (5 μL) was added to the wells and mixed by gentle swirling. The solutions were then incubated at 20 °C for 15 min away from light. The cells were stained with 10 μL of PI and incubated at 4 °C for 5 min in the dark. Finally, the apoptosis rate of the cells was detected by flow cytometry for 30 min. The FITC and PI were detected with 488 nm and 575 nm excitation light, respectively.

### DNA replication activity detection

5 Ethynyl 2 'deoxyuridine (EdU) is a thymidine nucleoside analogue that can replace thymine in replication during cell proliferation 27. Using the specific reactions between EdU and Apollo fluorescent dyes, the DNA replication activity of cells can be rapidly and accurately detected. EdU assays are faster, more sensitive, and more accurate than BrdU assays 28. Cells in logarithmic growth stage were inoculated in 96-well plates with 10^5^ cells per well and cultured to the normal growth stage for medical treatment. Each well received 100 μL of 50 μM EdU (Cell-Light^TM^ EdU Apollo643 *In vitro* Kit, RiboBio, Suzhou, China) at 1:1000 dilution in 15% cell medium, and the samples were then incubated at room temperature for 2 h. The culture medium was discarded, and the plate was washed twice with PBS. The cells were fixed at room temperature for 30 min with 4% paraformaldehyde. The stationary liquid was discarded, 50 μL of 2 mg/mL glycine was added to each well, and the samples were incubated in a decolourising shaker for 5 min. The glycine solution was then discarded, and the plate was washed with PBS for 5 min. Next, 100 μL of 0.5% Triton X-100 was added to each well and incubated for 10 min. Apollo staining solution (100 μL) was added to each well, and the samples were incubated at room temperature for 30 min away from light. The Apollo staining solution was discarded, and the plate was washed with 0.5% Triton X-100 for 30 min. Then, 100 μL Hoechst 33342 reaction mixture was added to each well and the solutions incubated at room temperature for 30 min away from light. The Hoechst 33342 reaction mixture was discarded, and the plate was washed with PBS three times. Ultimately, the cell staining results were observed under a laser confocal microscope (NIKON, Tokyo, Japan).

### Detection of knock-in efficiency

We constructed a protomerless DsRed2 reporter vector to determine the effect of small molecule inhibitors on homologous-directed repair (HDR) efficiency. For the FGF5 locus, the DsRed2 reporter vector consisted of a 926-bp 3'-homology arm (HA), a DsRed2 coding sequence, and a 1099-bp 5'-HA (Figure 2A). Twelve hours after electroporation transfection of GFFs, the small molecule inhibitors were added to the cell culture medium and treated for 24 h. After changing the normal culture medium, the culture continued for 24 hours and fluorescence-activated cell sorting (FACS) was used to analyse the proportion of red fluorescent cells (Figure 2C).

### Cell electroporation transfection

To avoid the toxicity of Lipofectamine to cells, the DsRed2 reporter vector, or the VEGF-targeted integration plasmid and Cas9/gRNA co-expression plasmid, were transfected into the GFFs by electroporation transfection (Super Electroporator NEPA21 *In vitro* & *In vivo* Electroporation, Nepa Gene, Chiba, Japan). The sequences and locations of target sites were shown in Figure 2F. The small molecule inhibitor-treated cells were adjusted to a concentration of 10^6^ cells per 100 μL. The electroporation transfection parameters were as follows: voltage 225 V, pulse length 2.5 msec, pulse interval 50 msec, number of pulses 4, decay rate 10%, and positive polarity. The cell suspension was transferred to an incubator with 5% CO_2_ at 37 °C.

### Production of knock-in cell colony

In consideration of the biosafety issues involved in gene-editing animals and the subsequent industrialisation of varieties, neither resistance screening nor fluorescent proteins were used in the screening of the knock-in cell colonies. To improve the efficiency of the integration of the *VEGF* gene and avoid the kanamycin resistance gene integrating into the cashmere goat genome, we used the restriction enzymes *SfiI* and *PciI* to linearise the *VEGF*-targeted integration plasmid, and recovered large fragments containing the upstream HAs, skin tissue specific-promoter keratin-associated protein (Kap) 6.1, the *VEGF* gene, PolyA, and the downstream HAs using agarose gel electrophoresis. After transfecting the main frame of the VEGF-targeted integration plasmid and the Cas9/gRNA co-expression plasmid into the GFFs, single cells were inoculated with flow cytometry into 96-well plates containing DMEM/F12 medium supplemented with 15% FBS. After approximately 15 days, the growth confluence of the single-cell clones reached 80-90%, and were transferred to 24-well plates for further culture. When the cells had grown to 80% in the 24-well plates, they were trypsinised. A part of the cell suspension was further cultured in the well, and the remaining was extracted for genotyping. The genomes of the cell colonies were extracted using the Wizard® Genome DNA Purification Kit (Promega, Madison, USA), following the supplier's protocol. The polymerase chain reactions (PCR) identification primer locations are shown in Figure 2A, and the sequences are shown in S1. Knock in cell colonies of the GFFs were selected as donor cells for subsequent SCNT.

### Off-target analysis

To ensure the safety of the gene-edited goats, we designed sgRNA for FGF5 exon 1, and predicted all the potential off-target sites (five mismatches within the 20-nucleotide sgRNA) of this sgRNA in the goat genome, using the CCTop-CRISPR/Cas9 target online predictor 29. We then conducted PCR and sequencing on the 10 sites with high off-target probability to detect whether off-target attachment occurred in those locations. The primer sequences are shown in S1.

### Somatic cell nuclear transfer and embryo transplantation

The ovaries of cashmere goats were mechanically dissected and the cumulus-oocyte complexes were collected and cultured in M-199 maturation medium (Hyclone) in an incubator with 5% CO_2_ at 38.5 °C for 18 h. The cumulus-oocyte complexes were digested in 0.1% hyaluronidase solution, and the cumulus cells on the surfaces of mature oocytes were completely removed. After the nuclei of the mature oocytes were removed, the donor cells were microinjected into the perivitelline spaces of the enucleated oocytes in cytochalosin B droplets. Subsequently, fusion was performed with ECM 2001 Electro Cell Manipulator (Harvard Apparatus, Cambridge, USA). The reconstructed embryos were pre-treated in synthetic oviductal fluid with amino acids (SOFaa solution) containing 5 M IA23187 for 5 min and then transferred to SOFaa solution containing 2 mM 6-dimethylaminopurine. The embryos were cultured in an incubator at 38.5 °C with 5% CO_2_ for 3.5 h for activation. The reconstructed embryos were then cultured in an *in vitro* development solution at 38.5 °C for 48 h. We selected embryos that had developed to the two-cell, four-cell, and eight-cell stages and surgically transferred them into the oviducts of recipient cashmere goats in oestrus, with each recipient receiving 3-4 embryos.

### Southern blot hybridization

For Southern blotting, 3 µg of DNA, extracted from cashmere goats and digested by *XhoI* and *NdeI*, was electrophoresed in 1.5% agarose gel and transferred to nylon membranes by capillary transfer. PCR amplification was performed using a VEGF-targeted integration plasmid as a template, and the probe fragment was purified after agarose gel electrophoresis. The location of the probe is shown in Figure 2A, and its primer sequences are shown in S1. The probe was combined with digoxigenin for hybridisation. Hybridisation was conducted at 42 °C for 16 h. The hybridised probes were immunodetected using anti-digoxigenin-alkaline phosphatase and visualised with the chemiluminescence substrate CSPD (Roche, Basel, Switzerland).

### Skin tissue paraffin sectioning & HE stain

The goat skin tissues were fixed in 4% paraformaldehyde for 24 h, washed under running water, dehydrated using graded ethanol, vitrificated with dimethylbenzene, embedded in paraffin, and sectioned (7 μm). After dewaxing the sections with xylene, they were rehydrated with ethanol and rinsed with distilled water to remove the residual xylene and ethanol. The cell nucleus was stained with haematoxylin for 10 min, and then the cytoplasm was stained with eosin for 30 s. After gradient dehydration with ethanol and dimethylbenzene, neutral balsam was added to the sections before covering them with a microscope coverslip.

### Western blotting

The Mammalian Protein Extraction Kit (CWBIO, Beijing, China) was used to extract cashmere skin proteins, and the concentration of the protein samples was measured using the PierceTM BCA Protein Assay Kit (Thermo Scientific) and a Varioskan Flash reader (Thermo Scientific, Thermo Scientific, Waltham, USA). Subsequently, sodium dodecyl sulphate-polyacrylamide gel electrophoresis and transmembrane analysis were carried out using PowerPac Basic (Bio-Rad, Berkeley, USA) and PowerPac HC^TM^ (Bio-Rad). Tris Buffered (CWBIO, Beijing, China) saline Tween was used to wash the nitrocellulose filter membrane for 5 min. After blocking the membranes with 5% skim milk (Difco^TM^ Skim Milk, BD Biosciences) for 2 h, alpha-tubulin antibody (Proteintech, Wuhan, China), FGF5 antibody (Proteintech, Wuhan, China), and VEGF antibody (Proteintech) were added at a dilution of 1:1000 in 5% bovine serum albumin, and the samples incubated at 4 °C overnight. After washing-off the excess primary antibodies with TBST, the secondary antibody (Horseradish peroxidase-conjugated affinipure goat anti-rabbit IgG, Proteintech) was added at a dilution of 1:1000 in 0.5% bovine serum albumin and the samples incubated for 1 h at room temperature. The membrane was washed three times with TBST to remove the secondary antibody. Finally, Pierce^TM^ ECL Western Blotting Substrate (Thermo Scientific) was used for colour rendering, and the Tanon-5200 image-analysis system (Tanon, Shanghai, China) was used to visualise the protein bands.

### Real-time polymerase chain reaction

Total RNA was extracted from the skin tissue of cashmere goats using RNAiso Plus (TaKaRa Bio, Shiga, Japan). The integrity and molecular weight of the RNA were checked using denaturing agarose gel electrophoresis. The RNA concentration was determined using a Nanodrop 2000 spectrophotometer (Thermo Fisher). The cDNA was prepared from 500 ng of RNA using the PrimeScript^TM^RT Reagent Kit (TaKaRa Bio), following the supplier's protocol. Quantitative real-time PCR was performed with SYBR Premix Ex Tap^TM^II (TaKaRa Bio), and the relative expression of the target gene was calculated following the 2^-ΔΔCt^ method. The names and sequences of the primers are provided in S2.

### Quality and yield of cashmere

Cashmere quality and yield are important factors for measuring economic benefits. All the cashmere of the three goats was collected and weighed. The fibre was smashed and its fineness measured using a BT Benchtop mass spectrometer (OFDA, Sydney, Australia). Fracture strength, breaking strength, and elongation at break were measured using the Yg006 Electronic Single fibre Strength Tester (Yanshuo, Xian, China). We used a fibre diagram machine (Wira Instrumentation, Bradford, England) to randomly select fibres to measure length.

### Multiple omics next generation sequencing

Skin tissues of cashmere goats were collected as samples for transcriptome, proteome, and metabolome studies. Eukaryotic mRNA sequencing was based on the Illumina Novaseq 6000 (Illumina, San Diego, USA) sequencing platform. The clean reads of each sample were compared with the designated reference genome. Based on the quantitative results of the expression levels, the differentially expressed genes (DEGs) between two groups were analysed. The difference analysis software used was edgeR, and the screening threshold was |log2FC| ≥ 0.848 & padjust < 0.05. In order to exclude the possible influence of somatic cell nuclear transplantation on individual goats, SCNT and WT were used as the control group and compared with GEC in the subsequent analysis.

The instrument used for proteome mass spectrometry was the Q_Exactive HF-X (Thermo Scientific). A UPLC-TripleTof system (AB SCIEX, WOODLANDS, Singapore) was used for liquid chromatography-mass spectrometry non-targeted metabolome sequencing. The Gene Ontology (GO) enrichment and Kyoto encyclopedia of genes and genomes (KEGG) enrichment were analysed and visualised on the free online Majorbio Cloud Platform.

### Statistical analysis

The results are presented as mean ± standard deviation (SD). Student's t-tests were carried out to analyse the differences in the measurement data. A P-value < 0.05 was taken to indicate statistical significance.

## Results

### RS-1 and Nu-7441 treatment enhances CRISPR/Cas9 mediated knock-in efficiency in GFFs

**Figure 1:**
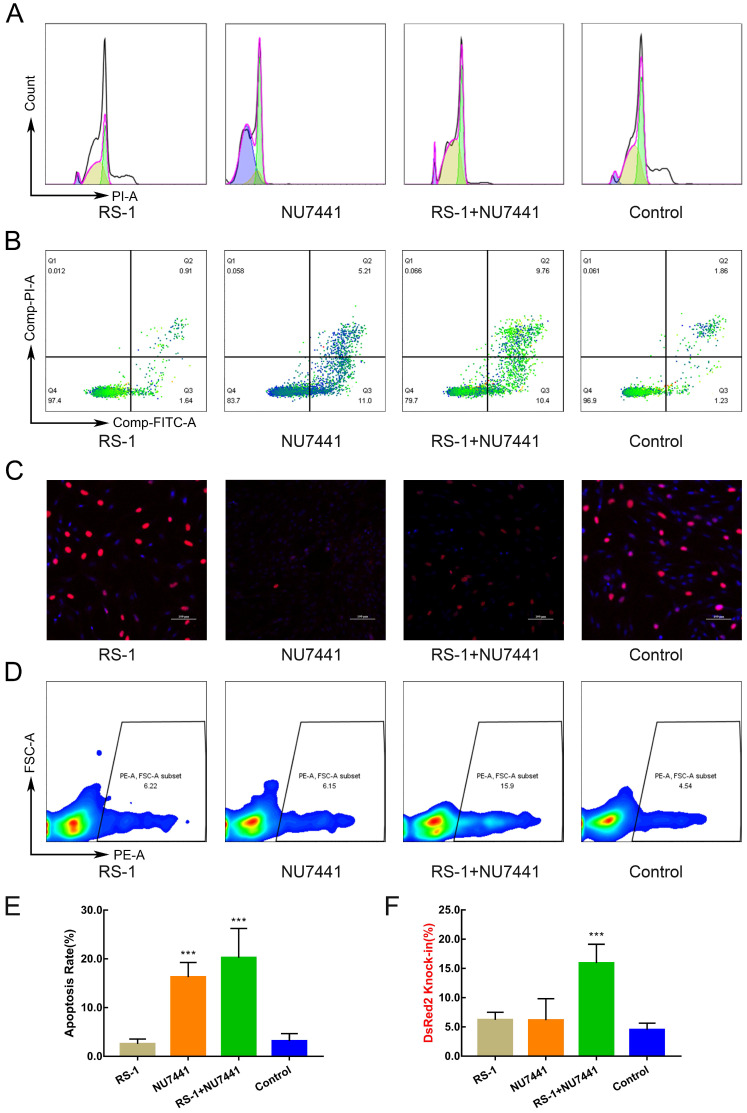
Effects of small molecules on cell cycle, apoptosis, proliferation, and HDR efficiency. **A.** Cell cycle after treatment with small molecules. **B.** Cell apoptosis. **C.** Cell proliferation and toxicity assay. **D.** HDR efficiency of DsRed2 knock-in. **E.** Cell apoptosis statistics. **F.** HDR efficiency statistics of DsRed2.

The cell colony of *VEGF* integrated at *FGF5* site was used for subsequent SCNT, which was derived from the proliferation of a single cell and required high cell status and quantity. Therefore, we hope to reduce the adverse effects on cells while improving HDR. Therefore, 24 h after the GFFs were treated with different small molecules, the cell cycle, apoptosis, and DNA replication activity were respectively detected. Using NU7441 alone had a great effect on the cell cycle (Figure 1A). In addition, NU7441 increased the number of apoptotic cells (Figure 1B, E). Because the inhibitors added were all related to DNA repair, we also examined DNA replication activity. Similar to apoptosis, RS-1 had the least effect on DNA replication (Figure 1C).

To ensure that the trends in the experimental results were consistent, the sequence and length of the HA of the DsRed2 reporter vector were identical to those of the *VEGF* site-directed integration vector. By means of FACS, we found RS-1 and Nu-7441 treatment increased HDR efficiencies by 3-4 times (Figure 1D, F) and had less effect on cell cycle, apoptosis, and DNA replication activity.

### Production of knock-in cell colony

**Figure 2:**
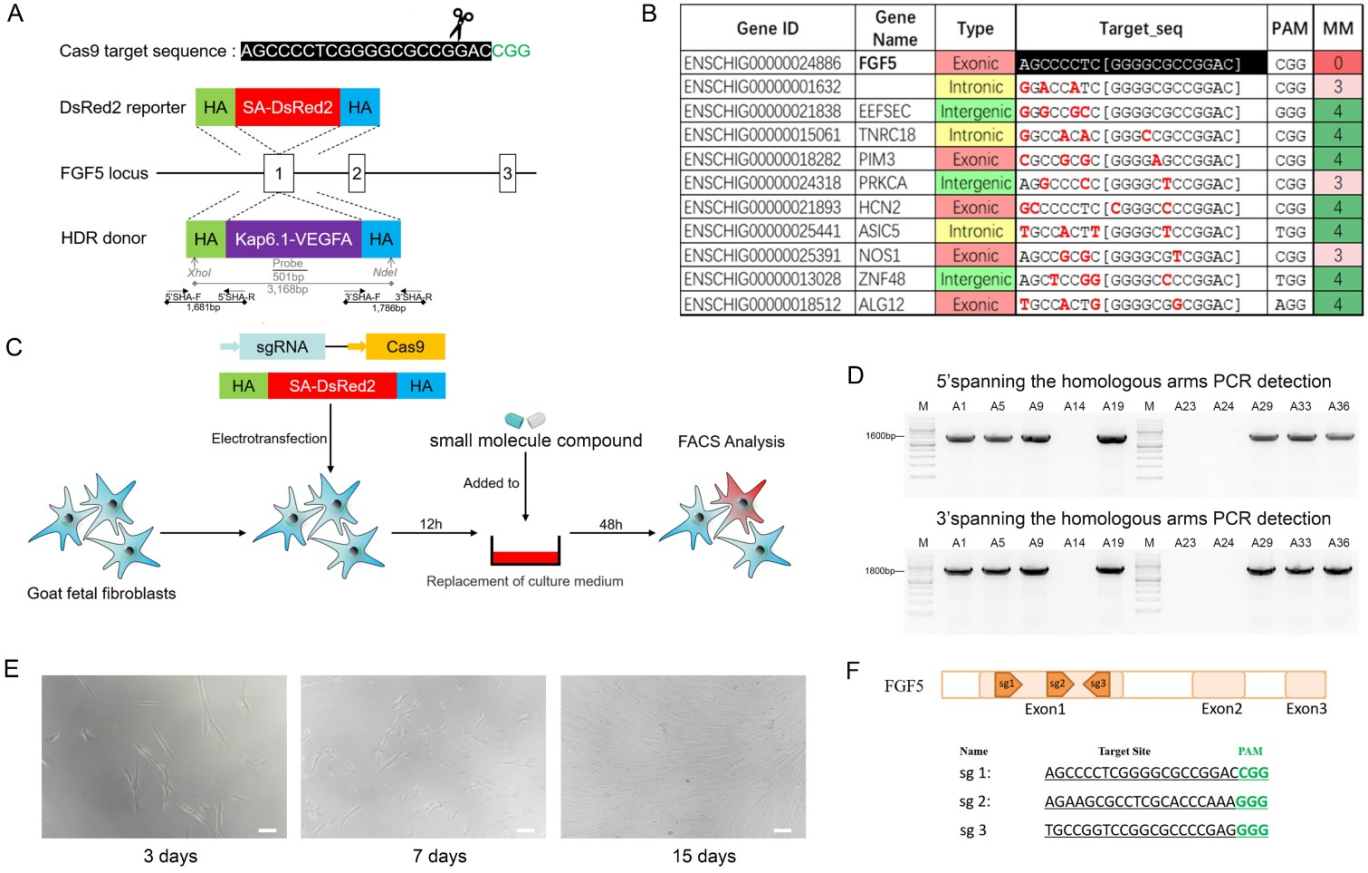
** Production of Knock-in cell colony. A.** Gene-targeting strategy of Cas9-mediated knock-in of VEGF or DsRed2 to the FGF5 locus. **B.** Gene names, types, and target sequences of the top 10 potential off-target sites of sgRNA were used in this study. **C.** Process of exploring the optimal HDR efficiency of small molecules. **D.** Spanning the homologous arms PCR identification results of cell colonies. **E.** Images of cell colonies at 3, 7, and 15 days. Scale bar = 100 µm. **F.** The sequences and locations of target sites at exon 1 of FGF5 gene.

Flow cytometry was used to inoculate the single cells into 96-well plates for culture. After approximately 15 days, the cells were subcultured and identified (Figure 2E). The genomic DNA of cell colonies was extracted for PCR identification, and the partial electrophoresis results were shown in Figure 2D. The sequencing results confirmed that seven cell colonies achieved VEGF knock-in. To ensure the efficiency of the targeted integration of the *VEGF* gene, we transferred the Cas9/gRNA co-expression plasmids into GFFs, instead of Cas9 mRNAs or proteins, which increased the risk of off-target attachment to some extent. PCR and sequencing of 10 potential off-target sites (Figure 2B) showed that none of them contained mutations.

### Effect of inhibitors treatment on embryo cleavage

**Figure 3:**
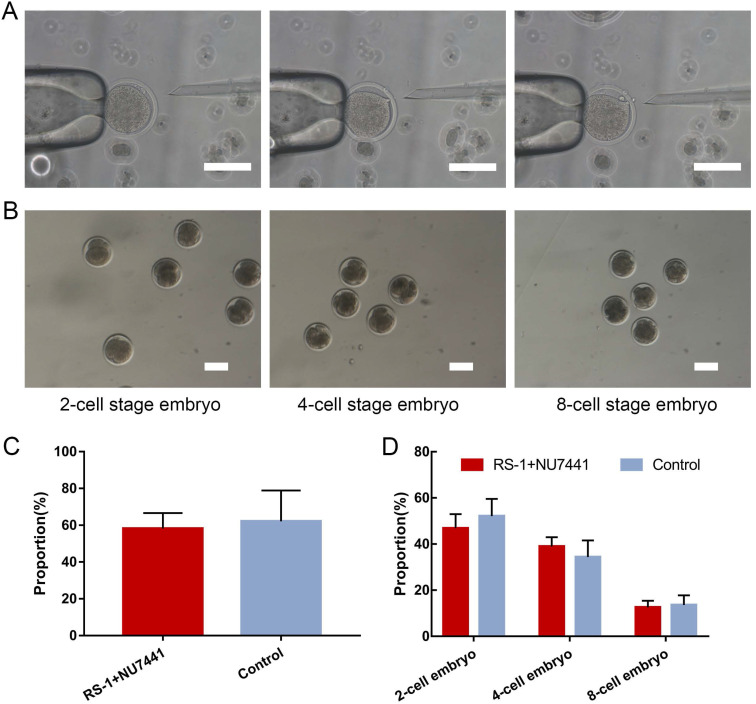
** Generation of gene-edited clone embryos. A.** Process of somatic cell nuclear transfer. Scale bar = 100 µm. **B.** Embryos at different stages of development. Scale bar = 100 µm. **C.** Cleavage rate of donor cells treated with RS-1 and NU7441. **D.** Ratio of the cloned embryos at different developmental stages from donor cells treated with RS-1 and NU7441.

A total of 1,824 oocytes were collected from 463 ovaries through mechanical cutting, and 936 mature oocytes were obtained after 18 h of *in vitro* culture, with a maturity rate of 51.3%. The mature oocytes were used for SCNT (Figure 3A). After 48 h, embryo cleavage had developed to different stages (Figure 3B). The donor cells used for SCNT have no significant effect in the embryo cleavage rates or percentages of embryos at different stages after being treated with small molecules (Figure 3C, D). The results showed that RS-1 and NU7441 treatment had no adverse effects on embryos.

### Acquisition of cloned cashmere goats

**Figure 4:**
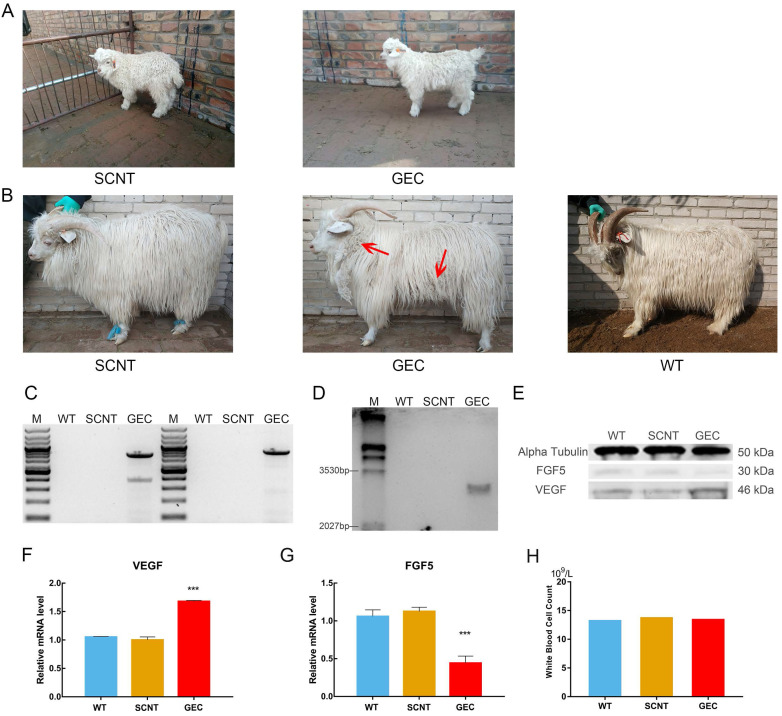
** VEGF knock-in Cashmere goats and its identification and detection. A.** Images of 1-month-old SCNT and GEC. **B.** Images of 15-month-old SCNT, GEC, and WT. Red arrows indicate tufts of cashmere. **C.** Spanning the homologous arms PCR identification results of WT, SCNT and GEC. **D.** Southern blot identification results of SCNT, GEC, and WT. **E.** VEGF and FGF5 expression in skin tissues of WT, SCNT and GEC by Western blot. **F, G.** RT-PCR was used to detect the expression of VEGF and FGF5 in skin tissues of S WT, SCNT and GEC. **H.** Routine blood tests were performed on WT, SCNT and GEC, and the number of WBC was counted.

There were two types of donor cells in the SCNT: mutated cell colonies with *VEGF* integration in the *FGF5* site and GFFs from the same source of embryos without gene editing. A total of 97 2-cell embryos, 73 4-cell embryos, and 25 8-cell embryos were obtained when the gene-edited cells were used as donor cells. They were transplanted into 69 recipient goats, and after approximately 150 days of gestation, a cashmere goat named gene-edited clone (GEC) was born on March 4, 2018 (Figure 4A). A total of 75 2-cell embryos, 70 4-cell embryos, and 21 8-cell embryos of non-gene-edited cells were obtained as donor cells. They were transplanted into 62 recipient goats, and a cashmere goat named SCNT was born on February 23, 2018 (Figure 4A).

### Characterisation of VEGF knock-in cashmere goat

After GEC and SCNT were born, their blood samples were collected for genome extraction, and PCR and electrophoresis were performed. Spanning the upstream and downstream homologous arms PCR results as shown in the Figure 4C, and the Sanger sequencing was correct. To further verify the PCR results, we performed Southern blot analysis. The genomes of GEC, SCNT, and wild type (WT) were treated with the restriction enzymes *XholI* and *NdelI*, and exposed after electrophoresis, membrane transfer, and hybridisation. Only GEC showed a specific band of 3,168 bp (Figure 4D). The results of PCR and Southern blot indicated that GEC realised the integration of *VEGF* at the *FGF5* site. We then measured the expression levels of *VEGF* and *FGF5* in skin tissues of cashmere goats. The results showed that the expression level of *VEGF* was upregulated at both the transcriptional and protein levels, whereas the expression level of *FGF5* was downregulated (Figure 4E-G).

**Figure 5:**
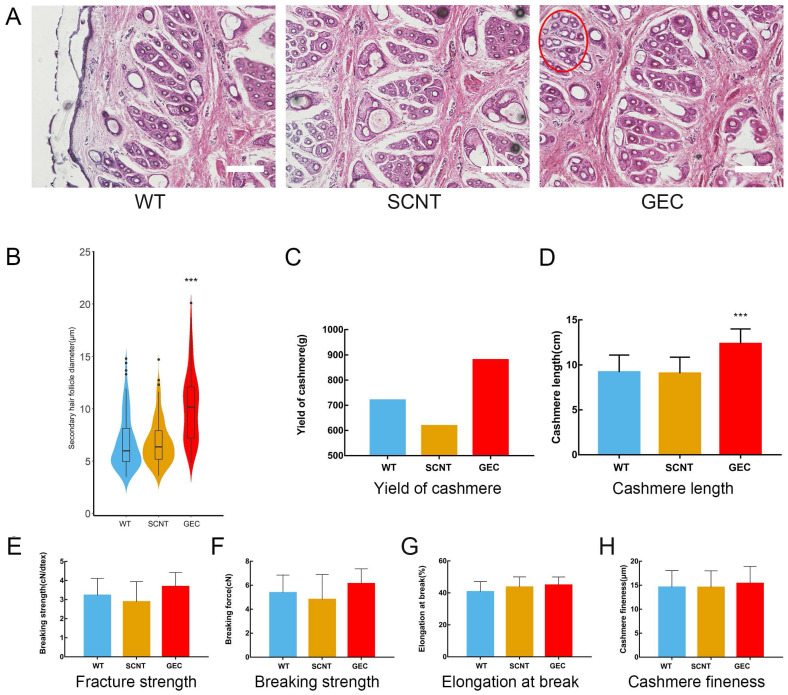
** Skin tissue sections and cashmere quality detection. A.** Paraffin sections and H&E staining of WT, SCNT and GEC. Diameter of the secondary follicle in the red circle increased. Scale bar = 100 µm. **B.** Secondary hair follicle diameter statistics of WT, SCNT and GEC. **C-H.** Cashmere yield, cashmere length, fracture strength, breaking strength, elongation at break, and cashmere fineness, respectively, of WT, SCNT and GEC.

No difference from other cashmere goats was observed in GEC at the age of 1 month. At the age of 15 months, after a hair follicle growth cycle, the cashmere synthesis of GEC was more vigorous than those of SCNT or WT, and clusters of cashmere could be clearly seen on the shoulders, neck, and abdomen (Figure 4B). Histological analysis revealed that the secondary hair follicle diameter of GEC seemed to have increased (Figure 5A). We found that it was significantly increased after statistical analysis of different sections (Figure 5B).

In addition, previous work suggested the overexpression of VEGF in the skin caused inflammation 30. We were also very concerned about the health of gene-edited cashmere goat, so the blood routine test was carried out, and the white blood cell count was shown in Figure 4H. The reference value of white blood cell count in goat is 11.32-15.08×10^9^/L. This result suggested that VEGF does not cause inflammation in the gene-edited goat.

### Determination of yield and quality of cashmere

The value of cashmere depends greatly on the length and fineness of its fibres. Therefore, we measured the related indexes of the GEC cashmere. After collecting and weighing the cashmere, the GEC cashmere yield seemed to be higher than the SCNT or WT cashmere yields (Figure 5C). However, as only 1 year's data were collected, no one-way ANOVA was conducted. The cashmere fibre length of GEC hairs had increased by approximately 30% (Figure 5D). The results indicated that the downregulation of FGF5 prolonged the growth period of hair follicles and increased the length of the cashmere. The mechanical properties of cashmere are important to the textile industry, so we examined the fracture strength, breaking strength (Figure 5E-H), elongation at break, and fineness of the GEC cashmere, and found no significant differences from those of the SCNT and WT cashmere. The above results showed that the increase in yield and fibre length of the cashmere produced by GEC did not affect its quality.

### Overview of transcriptome data

**Figure 6:**
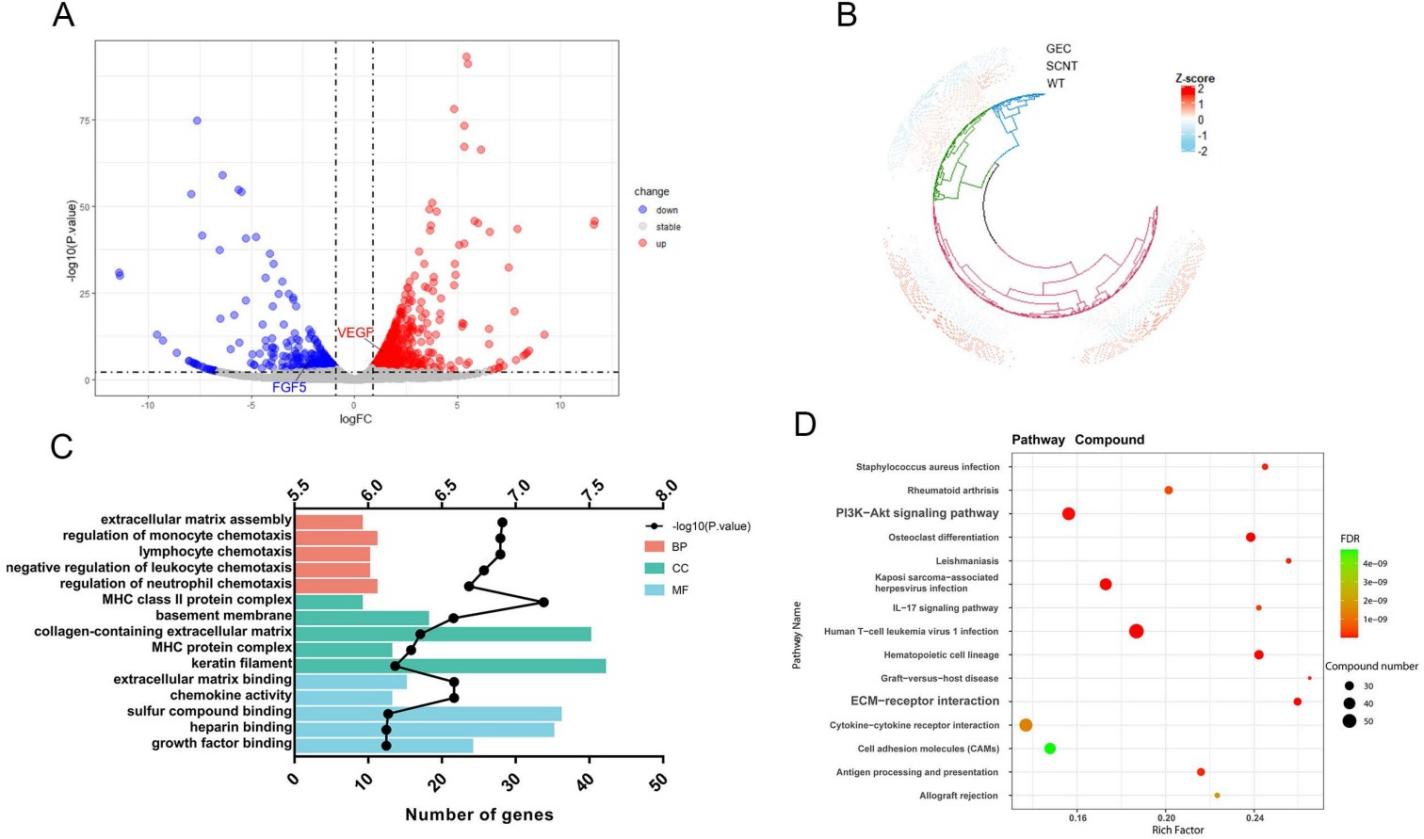
** Skin transcriptome sequencing result of cashmere goats. A.** Volcano diagram of transcriptome sequencing. The X-axis is the log2FC value, and the Y-axis is -log10 (P. value). There were 843 genes upregulated and 403 genes downregulated in GEC. **B.** Cluster analysis of gene expression patterns was performed for all detected genes. **C.** GO enrichment analysis was performed for all the differentially expressed genes. The Y-axis is the GO term. The top X-axis is -log10 (P. value) and the bottom X-axis is the number of genes in each GO term. **D.** KEGG enrichment analysis was performed on the differentially expressed genes. We show 15 KEGG pathways with minimum FDR values. The size of the dot represents the number of genes in each pathway.

To reveal the mechanism by which *VEGF* and *FGF5* promote hair growth, transcriptome, proteome, and metabolome studies were conducted on goat skin tissues. Transcriptomic analysis of the three samples was completed, and a total of 22.33 Gb of Clean Data was obtained. The Clean Data of each sample reached more than 6.59 Gb, and the percentage of the Q30 base was over 94.01%. The sequence alignment rate ranged from 95.02-96.1%. In this analysis, a total of 22,167 expressed genes were detected, including 19,266 known genes and 2,901 new genes. Finally, 843 genes were upregulated and 403 genes were downregulated. Volcano mapping was performed on all the detected genes, and *VEGF* and *FGF5* were both DEGs (Figure 6A). A cluster analysis of the 1,246 DEGs showed that the clusters of SCNT and WT were intact (Figure 6B). Gene ontology enrichment analysis was performed on all the DEGs, and 24 genes were enriched in growth factor binding and extracellular matrix (ECM) assembly functions (Figure 6C). Consistent with this, KEGG revealed that the DEGs were significantly enriched in the PI3K-AKT signalling pathway and ECM-receptor interactions (Figure 6D). These results suggested that *VEGF* and *FGF5* altered cell growth status by affecting ECM-receptor responses and cell growth factor binding.

### DEGs enriched in the PI3K-Akt signaling pathway and ECM-receptor interaction

**Figure 7:**
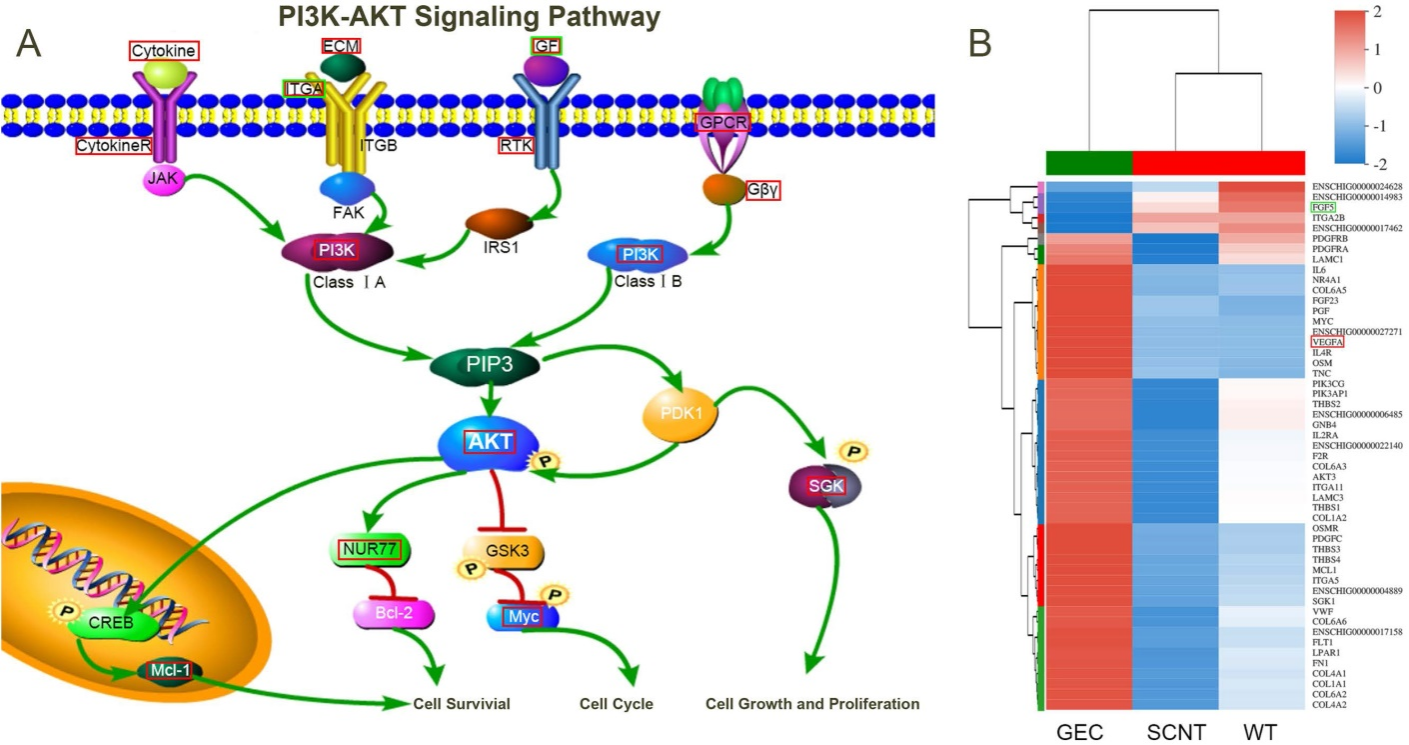
** PI3K-AKT Signaling Pathway. A.** Schematic diagram of key factors in the PI3K-AKT signalling pathway. **B.** Cluster analysis was performed on the genes enriched in the PI3K-AKT signalling pathway. The red box indicates upregulated, and the green box indicates downregulated genes.

A total of 51 DEGs were enriched in the PI3K-AKT signalling pathway. A clustering heat map was drawn for these 51 DEGs (Figure 7B) and the pathway was visualised (Figure 7A). In the figure, the red boxes represent upregulated gene expression, and the green boxes represent downregulated gene expression. Changes in the expression levels of cell membrane receptors, such as CytokineR, ITGA, RTK, and GPCR, and their ligands caused a cascade of intracellular amplification of signals acting on the key molecule, AKT. Finally, cell growth, proliferation, cycle, and survival were regulated by downstream McL-1, Bcl-2, Myc, and SGK.

**Figure 8:**
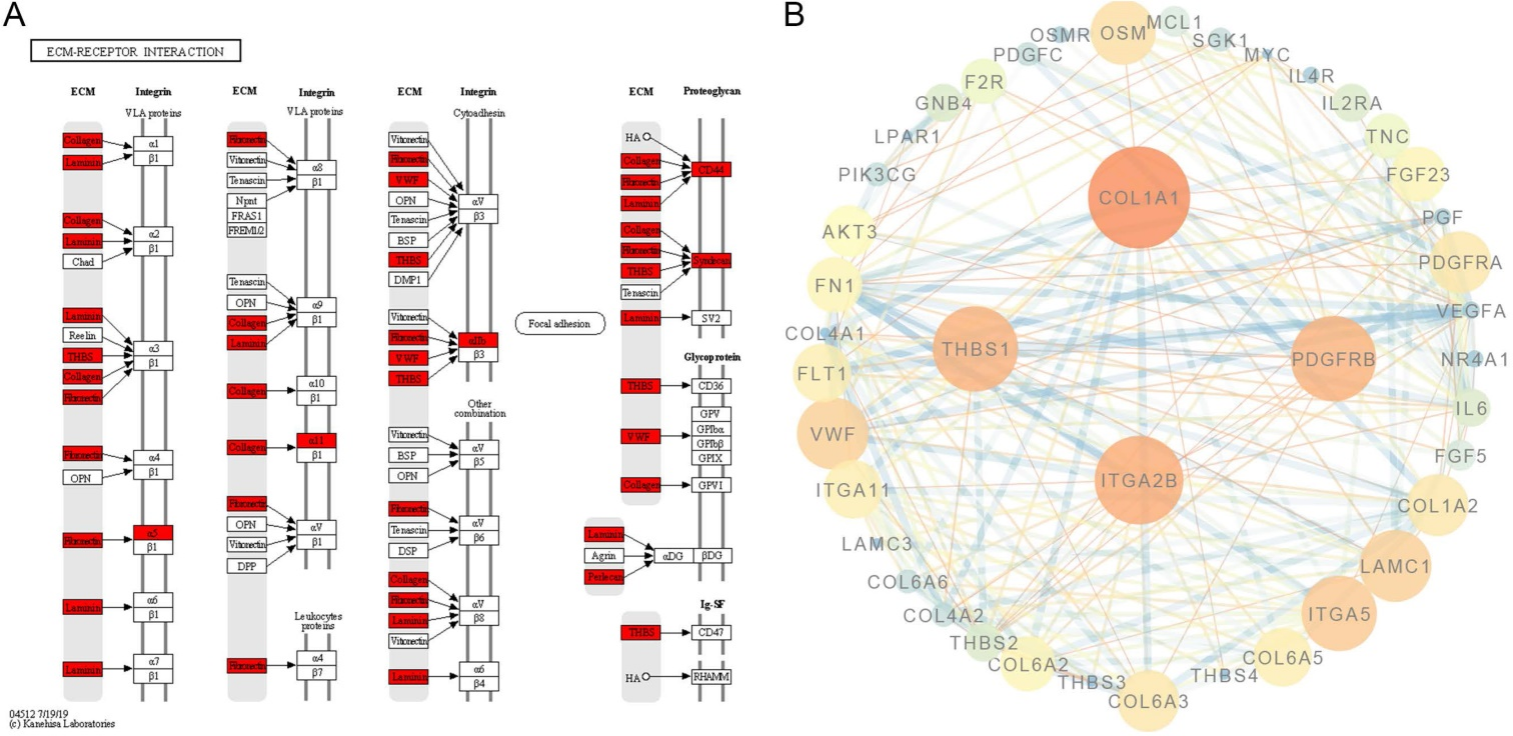
** Protein-protein interaction in the PI3K-AKT signalling pathway and ECM-receptor interactions. A.** ECM-receptor interaction pathway was adapted from KEGG; the red box indicates the upregulated genes. **B.** Protein-protein interaction network of genes enriched in the PI3K-AKT signalling pathway and ECM-receptor interactions. The core proteins were FN1, VEGFA, COL1A1, IL6, CD4, and PDGFRB.

In addition, DEGs were also enriched in ECM-receptor interactions. These were mainly ligands located in the ECM (Figure 8A). We visualised the protein-protein interaction network for the DEG-translated proteins enriched in the PI3K-AKT signalling pathway and ECM-receptor interactions, and the core proteins were fibronectin-1 (*FN1*), *VEGFA*, collagen type I alpha 1 (*COL1A1*), interleukin-6 (*IL6*), cluster of differentiation-4 (*CD4*), and platelet-derived growth factor receptor beta (*PDGFRB*) (Figure 8B).

### Correlation analysis of proteome and metabolome

**Figure 9:**
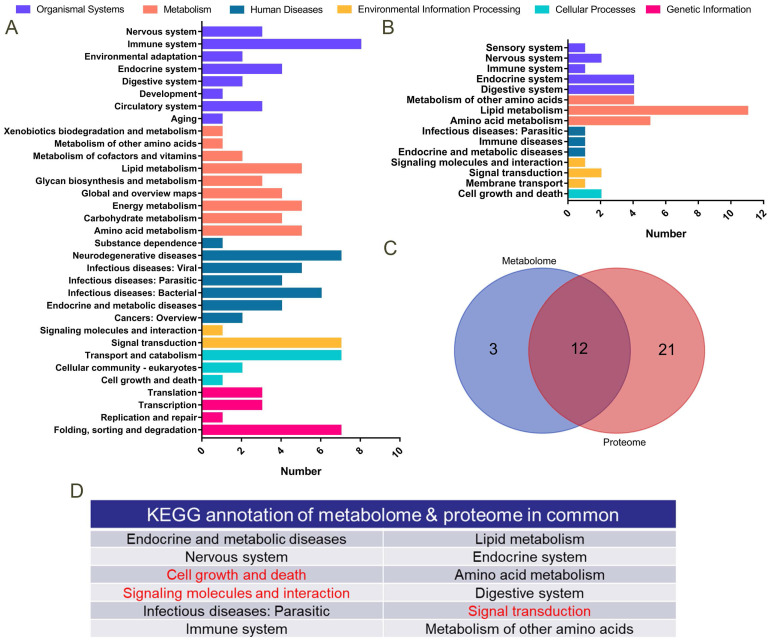
** Proteomics and metabolomics analysis. A.** KEGG functional annotation was performed on 178 differentially expressed proteins in proteomics sequencing. The Y-axis is the name of the KEGG metabolic pathway, and the X-axis is the number of proteins annotated to this pathway. **B.** KEGG functional annotation was performed on 122 differentially expressed metabolites in metabolome sequencing. **C.** Venn diagrams for the annotated pathways in metabolome and proteome sequencing. **D.** Table of 12 KEGG pathways annotated both in proteome and metabolome sequencing.

A total of 178 differentially expressed proteins was detected by proteomic sequencing, and KEGG functional annotation was performed (Figure 9A). A total of 1,706 differential metabolites was detected in the metabolome (122 of which were named), and KEGG functional annotation was performed (Figure 9B). A Venn diagram was drawn for the pathways annotated to the differential proteins and metabolites (Figure 9C), and 12 common annotated pathways were obtained (Figure 9D). Among them, cell growth and death, signalling molecules and interactions, and the signal transaction profile verified our transcriptome sequencing results.

## Discussion

DSB causes endogenous repair mechanisms in cells, such as non-homologous end joining (NHEJ), and HDR 31. NHEJ is the forced joining of two fractured DNA strands, and the repair process results in the insertion or deletion of nucleotide fragments, which destroys the open reading frame of the gene at the junction and produces mutations. Destroying the functional components of a gene or genome can help us to uncover its functions and mechanisms 32. The precise insertion or deletion of exogenous genes or regulatory sequences can be realised using HDR, which is the most dependable repair mechanism for genome site-specific integration 33.

The probability of cells using HDR for DNA repair is low (approximately 0.5% to 20%, depending on a variety of factors including cell type and cell state), whereas the probability of antagonistic NHEJ can be as high as 80% 34, 35. Therefore, we used small molecules to improve the efficiency of gene knock-in by promoting HDR and inhibiting NHEJ.

There have been many studies on small molecules promoting HDR efficiency, but the performance of the same inhibitor differs among different species and cell types 36. Here, the HDR activator RS-1, and NHEJ inhibitor NU7441 were selected for the experiment. We found that the efficiency of HDR was significantly improved when RS-1 and NU7441 were used together.

Although SCNT is the only known method of obtaining the totipotency for somatic cells so far, owing to various epigenetic disorders in reprogramming, the reprogramming of somatic cells is not complete, resulting in extremely low developmental potential for nuclear transplantation embryos 37. We found that RS-1 and NU7441 treatment of GFFs did not affect the embryo cleavage rate or ratio of cloned embryos at different developmental stages when the treated cells were used as donor cells. Therefore, RS-1 and NU7441 can be used in the generation of genetically edited goats.

However, only two goats were born from 131 recipients, and the live birth rate was only 1.5%. We speculate that this might be caused by the incomplete reprogramming of donor cells, resulting in the retardation or failure of embryo development in the early stage of transplantation to recipients. During preparation of the world's first somatically cloned monkey, the mRNA36 of histone lysine demethylase 4D was injected into the fused, activated, and reconstructed embryo to remove some of the key epigenetic modifications in the donor nuclear genes, significantly improving reprogramming efficiency 38. In subsequent experiments, we can also improve the efficiency of reprogramming and remove epigenetic modifications to improve the birth rate of individuals.

The safety of gene-edited organisms is controversial. While pursuing efficiency and practicality, researchers are increasingly concerned with the possibility of causing harm to human beings and the ecological environment. From the perspective of biological safety, we did not introduce resistance genes or fluorescent proteins in our preparations. In addition, we linked the KAP6.1 promoter upstream of the *VEGF* gene. KAPs are a class of hair follicle-specific proteins 39, and their promoters lead to the follicle-specific expression of downstream genes 40. In this study, the KAP6.1 promoter was used to initiate the transcription of *VEGF* in hair follicles, which not only promoted the development of cashmere follicles but also avoided the expression of VEGF in other tissues, which might have affected other physiological activities.

We found that VEGF expression was upregulated and *FGF5* was downregulated in the skins of cashmere goats. This means that we achieved the goal of *VEGF* overexpression and *FGF5* knockout. *FGF5* downregulation prolonged the anagen phase of hair and increased the length of cashmere fibres produced by GEC. Meanwhile, upregulated *VEGF* expression promoted the proliferation of cells that formed secondary hair follicles and increased their diameter. Furthermore, vigorous cashmere synthesis was promoted and clustering characteristics improved. Ultimately, cashmere yield was greatly improved without affecting its quality.

Previous studies have shown that *VEGF* can induce the differentiation of hair follicle stem cells into endothelial cells, thus promoting angiogenesis 13. It has been reported that after *FGF5* knockout, the ratio of secondary hair follicles (SHF) to primary hair follicles (PHF) and the length of cashmere fibres increased 24. In our gene-edited goat, we did not find an increase in the SHF/ PHF ratio, despite the fact that we statistically analysed a large number of histological sections. Since the specific molecular mechanism remained unclear, we used high-throughput sequencing technology to study it.

We found that *FN1*, *VEGFA*, *COL1A1*, *IL6*, *CD4*, and *PDGFRB* were the key components in the protein-protein interaction network. FN1 is involved in cell adhesion and migration processes, including embryogenesis, wound healing, blood coagulation, host defence, and metastasis 41. *COL1A1* is a fibril-forming collagen found in most connective tissues and is abundant in bone, cornea, dermis, and tendon tissues. *COL1A1* is associated with a particular type of skin tumour (dermatofibrosarcoma protuberans) resulting from unregulated expression of growth factors 42. *IL6* is primarily produced at sites of acute and chronic inflammation, where it is secreted into the serum and induces a transcriptional inflammatory response through *IL6* receptor alpha 43. *CD4* is a cell-surface glycoprotein involved in cell-cell interactions and cell adhesion and migration 44. These key molecules are involved in cell interactions, adhesions, and migrations and promote the synthesis of cashmere.

In conclusion, we found that a combination of RS-1 and NU7441 could improve the efficiency of CRISPR/Cas9-mediated HDR, without affecting the embryo status. Based on these findings, we obtained a cashmere goat that integrated the *VEGF* gene at the *FGF5* site. The diameter of the secondary hair follicles and the length of the cashmere fibres of this gene-edited cashmere goat were improved. The upregulation of *VEGF* and the downregulation of *FGF5* affected cell cycle, proliferation, and vascular tone through the PI3K-AKT signalling pathway, ECM-receptor interactions resulted in the gene-edited cashmere goat showing impressive cashmere performance. The results of this study provide a basis for the efficient generation of gene knock-in animals. It also provides an animal model for the study of the hair growth-promoting mechanisms in cashmere goats.

## Supplementary Material

Supplementary tables.

## Abbreviations

VEGF: vascular endothelial growth factor
FGF5: fibroblast growth factor 5
DSB: double-strand break
CRISPR: clustered regulatory interspaced short palindromic repeat
SCNT: somatic cell nuclear transfer
GFFs: goat foetal fibroblast cells
DMEM: dulbecco's modified eagle's medium
HDR: homologous-directed repair
HA: homology arm
FACS: fluorescence activated cell sorting
Kap: keratin-associated protein
PCR: polymerase chain reactions
DEGs: differentially expressed genes
GO: Gene Ontology
KEGG: Kyoto encyclopedia of genes and genomes
SD: standard deviation
GEC: gene-edited clone
ECM: extracellular matrix
FN1: fibronectin-1
COL1A1: collagen type I alpha 1
IL6: interleukin-6
CD4: cluster of differentiation-4
PDGFRB: platelet-derived growth factor receptor beta
NHEJ: non-homologous end joining
