# Supplementary material for: Generation of VEGF knock-in Cashmere goat via the CRISPR/Cas9 system
Source: Int J Biol Sci. 2021 Mar 2;17(4):1026–40. doi: 10.7150/ijbs.55559 (PMC8040296; doi:10.7150/ijbs.55559)
Supplement: Supplementary file 1 — Supplementary tables. [file ijbsv17p1026s1.pdf]

Supplementary materials

S1 PCR Primer

Primer name	Sequence
PROBE-F	AGCCAATGGCATGAAGGTGT
PROBE-R	TGCTGGCTTTGGTGAGGTTT
ENSCHIG00000001632-F	CGGGCTTTATACTTTAGTTC
ENSCHIG00000001632-R	GGAATGGAGGCACCGCAGTG
EEFSEC-F	CGCTGCAAACGCTGCCGA
EEFSEC-R	GGCAGCGGCACGCAGAAGC
TNRC18-F	CACGGGAGAAGCGGGTGGGG
TNRC18-R	CAGCACCTCCCGTGGGGCAC
PIM3-F	GCGTGTGAGCGCCTCGGCCT
PIM3-R	TGGCTGCGGGGAACCCAC
PRKCA-F	CGGCGGGCACCAATTGAGGA
PRKCA-R	GGAAGGAGGAGTCCAGGAGG
HCN2-F	TGCCCGCCTCGTCCGCCGGC
HCN2-R	TCCGCACTGCCCGGCGCCGC
ASIC5-F	AGTAATTTGGGTCTCTTTGA
ASIC5-R	TTTAGTTGCTAAGTGGTGTC
NOS1-F	GCCGGCTGGGGAGGAGATCG
NOS1-R	CGGGGCGGACGGGGTGAGCC
ZNF48-F	AGCCCAGGAAACACACGGTT
ZNF48-R	AGGAGACAGACACAATTTC
ALG12-F	TCTGAAACCCCAGCAGGCGC
ALG12-R	GTTCCGCTGTCCGTCCGCAG
5’SHA-F	TGATGAAGTTCATGGATGTCT
5’SHA-R	AGTCTGAGCAAAATGCATTAT
3’SHA-F	TGATGAAGTTCATGGATGTCT
3’SHA-R	TGTTGCCTCGGAGAGACA

S2 Q-PCR Primer

Primer name	Sequence
GAPDH-F	CGTGTCCGTTGTGGATCTGA
GAPDH-R	GAGTGTCGCTGTTGAAGTCG
FGF5-F	TCAGCACGTCTCTACCCACTTT
FGF5-R	CTTGACGGGATTAGGTGGTTTT
VEGF-F	TGTAATGACGAAAGTCTGGAGTGTG
VEGF-R	ATCTCTCCTATGTGCTGGCTTTG
